# The progress of protein synthesis factors eIFs, eEFs and eRFs in inflammatory bowel disease and colorectal cancer pathogenesis

**DOI:** 10.3389/fonc.2022.898966

**Published:** 2022-10-31

**Authors:** Conggai Huang, Qi Zhao, Xiaoqing Zhou, Ran Huang, Yi Duan, Johannes Haybaeck, Zhihui Yang

**Affiliations:** ^1^ Department of Pathology, The Affiliated Hospital of Southwest Medical University, Luzhou, China; ^2^ Department of Pathology, Basic Medical College of Southwest Medical University, Luzhou, China; ^3^ Department of Pathology, Neuropathology and Molecular Pathology, Medical University of Innsbruck, Innsbruck, Austria; ^4^ Diagnostic and Research Institute of Pathology, Medical University of Graz, Graz, Austria

**Keywords:** colorectal cancer, inflammatory bowel disease, eukaryotic gene expression, protein translation, colorectal pathogenesis

## Abstract

Colorectal diseases are threatening human health, especially inflammatory bowel disease (IBD) and colorectal cancer (CRC). IBD is a group of chronic, recurrent and incurable disease, which may affect the entire gastrointestinal tract, increasing the risk of CRC. Eukaryotic gene expression is a complicated process, which is mainly regulated at the level of gene transcription and mRNA translation. Protein translation in tissue is associated with a sequence of steps, including initiation, elongation, termination and recycling. Abnormal regulation of gene expression is the key to the pathogenesis of CRC. In the early stages of cancer, it is vital to identify new diagnostic and therapeutic targets and biomarkers. This review presented current knowledge on aberrant expression of eIFs, eEFs and eRFs in colorectal diseases. The current findings of protein synthesis on colorectal pathogenesis showed that eIFs, eEFs and eRFs may be potential targets for CRC treatment.

## Introduction

Colorectal cancer (CRC) is the third highest morbidity rate worldwide. It is also the second most common cause of cancer-related death (1). The incidence and fatality rate of CRC had increased in recent years, especially in developing countries (2). Even though many CRC patients are diagnosed early and undergo therapeutic surgery, chemotherapy and radiation therapy, the metastases and relapses still occurred in many patients (3, 4).

Inflammatory bowel disease (IBD) is an idiopathic, chronic inflammatory disorder of uncertain etiology with an underlying genetic predisposition. It includes Crohn’s disease (CD) and ulcerative colitis (UC) (5, 6). Although some intrinsic factors, such as host genetics, dysregulated immune responses, and microbial dysbiosis have been identified, the pathogenesis of IBD remained unclear (7–9). Nevertheless, there are unequivocal evidences of an association between IBD and CRC. A chronic inflammatory process is one of the responsible causes for the development of CRC (10).

Protein translation comprises several steps: initiation, elongation, termination and ribosomal recycling (11). Eukaryotic initiation factors (eIFs) played an essential part in the initiation of translation. The eIF signaling cascade is mainly influenced by the PI3K/AKT/mTOR pathway, regulating cell growth and proliferation (12–14). Eukaryotic elongation factors (eEFs) are active during protein chain elongation. They had been reported to be aligned by aminoacyl-tRNAs *via* their specific codons in mRNA (decoding), peptide bond synthesis, and movement of the mRNA associated with ribosome translocation. eEF1α, eEF1βγ and eEF2 facilitate these processes on the ribosome. Finally, the termination process is a release of the completed polypeptide chain, which requires eukaryotic release factors (eRFs).

Deregulation of protein synthesis results in abnormal gene expression, possibly bringing about uncontrolled cell growth, cancer development and progression (15). Deregulation of translation is related to colorectal pathogenesis of IBD and CRC. Previous studies had shown that dysregulation of eIFs, eEFs and eRFs is associated with cancer progression and malignant transformation (16, 17). Here, we reviewed the current research findings about eIFs, eEFs and eRFs, to demonstrate that they may be potential targets for IBD and CRC treatment.

## Eukaryotic translation initiation factors

### Eukaryotic translation initiation factor 1

eIF1 is a universally conserved translation factor with 113 amino acid (AA) and an important intermediary for initiating codon recognition in negative regulation. eIF1a is a 144AA long protein encoded on the X chromosome. eIF1a together with eIF2, 3, 4A, 4B and 4F triggers preinitiation complex formation. eIF1 and eIF1a have synergistically effects. Translation initiation induced by eIF1 had been shown to occur independently of p53. eIF1 was found to be increased a series of disease risks, such as aneurysmal bone cyst (18), Parkinson’s disease (19), hepatocellular carcinoma (HCC) (20), breast cancer (21) and ductal adenocarcinoma (22).

Enhanced expression levels of eIF1 indicates poor prognosis of CRC patients (14). eIF1 gene knockdown led to a significantly reduced proliferation rate and clonogenicity. eIF1 protein was overexpressed in low-grade and high-grade colon cancer (CC), and eIF4B protein was elevated in low-grade CC. The mRNA and protein expression level of eIF1 was significantly increased in rectum carcinoma (RC) tissues compared to normal colorectal mucosa tissues and low-grade CC. Although IBD is also closely associated with an increased risk of CRC, the relationship between eIF1 and IBD remained elusive.

### Eukaryotic translation initiation factor 2

eIF2 is a ternary complex involved in the formation of the eIf2-Met-tRNAi-GTP complex. At the initial stage of translation, the eIf2-GTP is hydrolyzed to eIF2-GDP. Then, the eIF2 gene, called eIF2AKs, is phosphorylated by four stress-responsive kinases. These include double-stranded heme-regulated kinase (HRI, also known as EIF2AK1), RNA-induced protein kinase (PKR, EIF2AK2), PKR like endoplasmic reticulum kinase (PERK, EIF2AK3) and control non-repressed two kinases (GCN2, EIF2AK4) at Ser51.

These four kinases phosphorylate eIF2 upon different events. eIF2B levels are lower than eIF2 in cells, so partial phosphorylation is sufficient to attenuate the initiation of protein synthesis. The mRNAs of a series of stress responses are resistant or stimulated by decreased the eIF2-GTP/Met-tRNAi ternary complex levels. The response is often been called the integrated stress response (ISR), and aberrant ISR has been linked to many human diseases (23, 24). eIF2a was been found differentially expressed in gastrointestinal cancer and lymphoma subtypes (25, 26).

**Figure 1:**
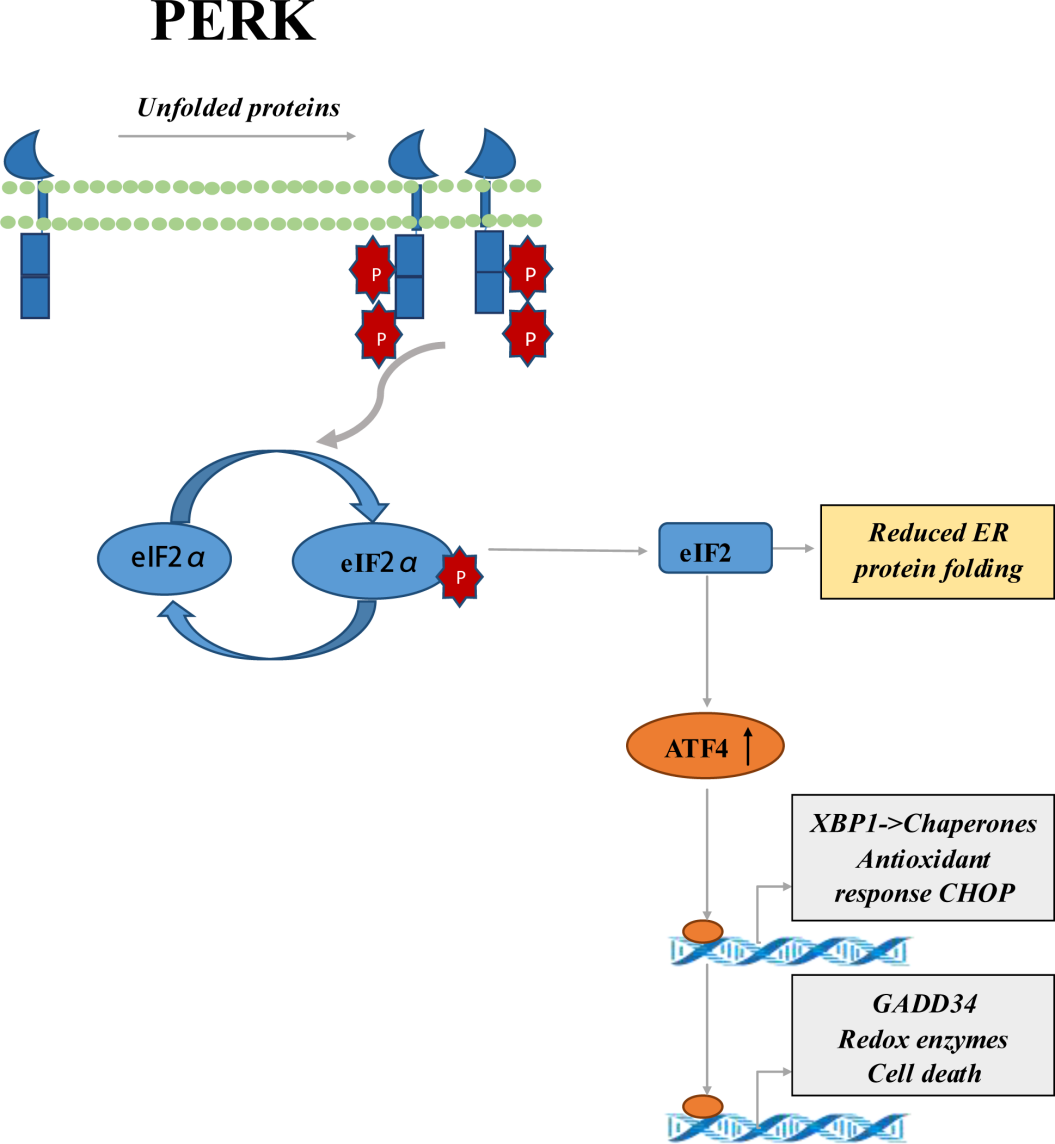
Schematic representation of PERK-eIF2α-ATF4 signaling pathway. It’s sensitive to ER stress, so PERK is activated. PERK activation leads to phosphorylation of the eIF2α ring, which inhibits protein translation. Phosphorylated eIF2α enhances the translation of ATF4, a transcriptional activator of genes associated with metabolism and nutrition, cellular redox status, and apoptosis regulation. ATF4 mediates the production of transcriptional factor CHOP, and the upregulation of CHOP can aggravate the development of colitis.

PRK was found to play a critical role in inflammasome activation, and interact with multiple inflammasome components, including the pyrin domain-containing 3 (NLRP3) of NLR family. The NLRP3 inflammasome is involved in the pathogenesis of IBD (27). Previous studies had shown that overexpression of EIF2AK2 could increase the activity of NLRP3 polymorphism during the development of IBD (28, 29). GCN2 is one of the vital coordinating factors of ISR. Researches reported that GCN2 has a protective effect on DSS-induced colitis in mice by inducing autophagy (30). Phosphorylated eIF2α had been reported to promote translation of the activating transcription factor 4 (ATF4) (31). PERK was known as one of the sensors of the ISR. Regulation of the PERK-eIF2α-ATF4 signaling pathway by inhibiting the dephosphorylation of eIF2α improves the clinical and histological effects of DSS-induced colitis in mice. PERK-eIF2α-ATF4 signaling pathway is a potential therapeutic target for IBD therapy (**Figure 1**) (28, 32). PERK activation had been shown to play an essential part in chemically induced apoptosis and contributes to G2/M arrest (33). The small-molecule PERK inhibitor may be used to activate the proapoptotic processin CRC cells (34). In addition, the study had shown that SPARC combined with GRP78 makes CRC cells sensitive to PERK/eIF2α and IRE1α/XBP-1 UPR signals by interfering with ER stress, resulting in the death of CRC cells (35). Smad7 knockout was related to inactivation of small eIF2 cells, decreased CDC25A expression, and partial reduction of proliferative cells in human CRC explants, as well as reduction numbers of intestinal tumors in Apc(min/+) mice (36).

### Eukaryotic translation initiation factor 3

eIF3 is the largest and most complex translation initiation factor in mammalian cells, with a molecular weight of about 550-800kDa. The eIF3 complex consists of 13 subunits, called EIF3A-M, and members of eIF3 family undertake various tasks during translation initiation.

The overexpression of eIF3a had been reported in several cancers, such as breast (37), lung (38), esophagus (39) and cervical cancer (40). eIF3a may play an important part in colon epithelial cell differentiation (41). The expression of eIF3a was also increased in CRC (42). eIF3a binds to phosphorylated eIF4b, facilitating the translation of IRES dependent proteins such as *myc*. The adenomatous polyposis *coli* (*APC*) gene mutations are tightly related to CRC (43). eIF3b was overexpressed in pancreatic Cancer (44), gastric cancer (45), as well as chronic myeloid leukemia (46). Overexpressed eIF3c was found in cholangiocarcinoma, lung adenocarcinoma and prostate cancer (47, 48). eIF3d is responsible for protein synthesis, which had been reported to play a carcinogenic part in CRC. eIF3d knockout significantly induced more HCT116 cells to accumulate in the sub-G1 phase, suggesting apoptotic cells increased after eIF3d knockout (49). eIF3e, also termed INT6, interacts with the interferon-induced protein P56. It reported that overexpression of eIF3e promoted CRC cell proliferation and decreased the overall survival of CRC patients (50). Decreased expression of eIF3e was also reported in breast (51) and lung cancer (52). eIF3f may be a vital regulator of cell migration, invasion, bioenergetics and metastasis. It is downregulated in cancers exemplified in melanoma, lung cancer and pancreatic cancer (53). A study showed that eIF3g is a targeted regulator of CRC chemotherapeutic resistance (54). A variant of eIF3h (rs16892766) was discovered to be associated with higher CRC risk (55). eIF3i is a proto-oncogene that causes CRC by directly upregulating the synthesis of COX-2 protein, activating the β-catenin/TCF4 signaling pathway (56). eIF3m is considered an indicator of poor prognosis in patients with CRC (57, 58).

### Eukaryotic translation initiation factor 4

eIF4 is a protein complex that promotes mRNA recruitment to a preassembled 43S preinitiation complex. eIF4 complex is composed of eIF4b and eIF4F complex, which is composed of eIF4a, eIF4e and eIF4g (59, 60). The presence of eIF4F complexe is critical for cap binding and subsequent RNA helicase activity that leads to protein translation. eIF4a is independent of the eIF4F complex, which stimulates eIF4a activity and promotes mRNA recruitment to the ribosome. eIF4b and eIF4e are regulated by the PI3K/AKT/mTOR signaling pathway. eIF4b activates S6K kinase, which is responsible for the phosphorylation of eIF4b at Ser-422120. The ability of eIF4E recognizing caps is regulated by binding to eIF4E binding proteins (4E-BP). Phosphorylation mediated by mTORC1 inhibited the activity of 4E-BP. When the binding of 4E-BP1 to eIF4e was weakened, subsequently the release of eIF4e was weakened (61–63). The result suggested that increased expression of eIF4E may be a vital factor for development of breast cancer (64). A case-matched and sex-matched transcriptome screening identified that eIF4E and eIF5 act as potential prognostic markers for male breast cancer (65). Studies had shown that targeting MUC1-C by GO-203 can inhibit the AKT-S6K-eIF4A pathway and block the proliferation and survival of CRC cells (66). Overexpression of eIF4e was reported to associated with poor prognosis in CRC patients (67, 68).

### Eukaryotic translation initiation factor 5

eIF5 is the GTPase activating protein (GAP) of eIF2 and plays a vital role in the initiation of translation, which may inhibit the guanine nucleotide exchange factor eIF2B (69). eIF5A, containing the unusual amino acid hypusine, which had been shown to stimulate ribosomal peptidyl activity and promote prolonged translation (70, 71). Overexpression of eIF5A may cause increased expression of p53 targets as well as p53-dependent apoptosis, so it was described as positive regulator of p53 (72).

In a recent study of Golob-Schwarzl and colleagues, overexpression of eIF5 was observed in CC and RC, and associated with survival rate (14). The overexpression of eIF5A had also been reported to be associated with poor prognosis in CRC (73). eIF5a was showed to induce apoptosis in CRC cells (HTC116 and HT29) and was associated with response of nucleus to tumor necrosis factor (TNF) signaling (74). The study had shown that upregulation of eIF5A2 could enhance epithelial mesenchymal transition (EMT) in CRC cells (HCT116 and HT29), and downregulation of eIF5A2 enhanced the chemosensitivity to doxorubicin in eIF5A2-positive cells (75). Deletion of eIF5B led an increase in ATF4 transcriptional translation through another mechanism. eIF5B silencing increased the expression of an ATF4-luciferase translational reporter by a mechanism requiring the repressive uORF2 (76). The ATF4 level was found reduced in the inflammatory intestinal mucosa of patients with IBD, so ATF4 plays a crucial role in maintaining intestinal homeostasis (77).

### Eukaryotic translation initiation factor 6

eIF6 is an anti-association factor in translation initiation, by binding to 60S subunits. It prevents premature connection of 40S and the interaction of 60S and 40S subunit, thus preventing the initiation of translation (78). eIF6 was phosphorylated by the complex of RACK1-PKCβII and thus cascaded by Ras. Another studies found that gene transcription of encoding eIF6 was regulated by the receptor Notch-1, which is a key downstream medium of oncogenic Ras (79–82). eIF6 was overexpressed in gallbladder cancer, head and neck cancer, non-small cell lung cancer and ovarian serous adenocarcinoma (12, 83–85), particularly in metastatic CRC (86). eIF6 plays a role in downstream protein synthesis of PI3K/AKT/mTOR (12). Overexpression of eIF6 increased cancer cell motility and invasion in CRC, in turn silencing of eIF6 significantly reduced the proliferation rate and the clonogenicity in HCT-116 CRC cell lines (12). A recent study by LJ found that eIF6 activated a variety of AKT-related cancer signaling pathways, such as p-AKT\MMP1\cyclinD1\Bcl2. Therefore, eIF6 could regulate cell proliferation, invasion, cell cycle and apoptosis under the background of CRC (87).

## Eukaryotic elongation factors

### Eukaryotic elongation factor 1

eEF1 is a complex factor composed of multiple subunits, responsible for binding to aminoacyl-tRNAs and transferring it to the A-site of ribosomes (88). Ras-driven cancers utilize methyltransfer-like 13 demethylations of eEF1A Lysine55 to increase translation output and promote tumorigenesis *in vivo* (89, 90). eEF1A1 and eEF1A2 can control cell motility, growth and death (91). Overexpression of eEF1A1 and eEF1A2 was related to a few different cancer types, such as plasmacytomas (92), HCC (93), clear cell renal cell carcinoma (94), breast cancer (95), gastric cancer (96), prostate cancer (97), ovarian cancer (98) and CRC (99). At the genomic level, a significant higher frequency of EEF1A2 copy number variation was found in patients with metastatic than non-metastatic CRC (99). Pellegrino found that EEF1A2 mediated the expression of PI3K/AKT/mTOR axis stabled oncogene MDM4 in HCC (100). eEF1G was found overexpressed in CRC (101).

### Eukaryotic elongation factor 2

eEF2 is answerable for the ribosomal translocation at the elongation stage of a polypeptide chain. Another important extension regulator is eEF2K. eEF2K is a Ca^2+^/calmodulin (CaM)-dependent kinase and a negative regulator of protein synthesis. eEF2 is overexpressed in lung cancer, esophageal squamous cell carcinoma, head and neck squamous cell carcinoma, pancreatic cancer, breast cancer, prostate cancer, non-Hodgkin’s lymphoma, melanoma, GBM and other human cancers (102, 103). It had been reported that in gastric cancer and CRC, overexpression of eEF2 could promote G2/M progression and cell growth *in vitro* and *in vivo* (104). Vasamsetti found that Muscarinic acetylcholine receptor (mAChRs) promoted the synthesis of the global protein of SNU-407 CC cells. mAChR-mediated dephosphorylation of eEF2 is regulated by the MEK1/2-ERK1/2 and the PKC pathway (105). mTORC1 gained power from eEF2K to promote translation elongation through S6K (106). mTORC1 had been shown to be an important downstream effector of Wnt signaling in the intestinal tract. The intestinal cell proliferation associated with Wnt signaling requires the mTORC1-S6K-eEF2K-eEF2 axis. eEF2K plays an important role in controlling the initiation of intestinal cancer and adenoma cell proliferation (107). eEF2K was downregulated in CRC, which was independently associated with poor overall survival in CRC patients (108). eEF2K acts as a tumor-suppressor in CRC. By contrast, it is established as an oncogene in other cancer entities like HCC, lung cancer, or triple-negative breast cancer (109).

## Eukaryotic releasing factors

The termination process of protein synthesis involves the hydrolysis of the final peptide-tRNA bond and the release of the nascent polypeptide. The reaction is mediated by eukaryotic releasing factor 1 (eRF1) and eRF3 proteins. In eukaryotes, when the eRF1-eRF3-GTP ternary complex binds to the termination codon of the ribosome A site, translation terminates (110). eRF1 is responsible for terminating protein biosynthesis by recognizing stop codon, binding ribosome and stimulating peptidyl-tRNA bond (111). eRF3 is a small GTPase that enhances the activity of eRF1. eRF3 is involved in key cellular processes, such as cell cycle regulation, cytoskeleton and apoptosis (112, 113). The N-terminal region of eRF3 contains polyglycine amplification encoded by a stable (GGC) channel in the *eRF3/GSPT1 exon 1* gene. Overexpression of *GSPT1* mRNA had been reported to be connected with gastric and breast cancer (114, 115). Malta-Vacas found that the GGC12 was present in 2.2% of CRC patients, but not in the CD cases (116).

Ribosome recycling usually occurs after a regular termination triggered by a termination codon. Recycling enables ribosomes and mRNAs to participate in more than one translation (117). ATPase ABCE1 had been shown to be a major ribosome recycling factor, while ABCE1-mediated post-TCs recycling is dependent on the presence of eRF1 at site A (118). eIF3 plays an important part in the post-TCs recycling of eukaryotic cells, promoting ribosome division into 40S subunits of 60S subunits and tRNA-and mRNA-bound after termination. eIF1 also mediates the release of tRNA at the P site, while eIF3j ensures subsequent mRNA dissociation (119).

## Conclusions

The occurrence and development of IBD are affected by many factors, such as host genetic susceptibility, intestinal flora, environmental factors and host immune system. Chronic inflammation may significantly increase the risk of cancer. Elevation levels of inflammation-related factors may also interfere with the control of cell proliferation.

eIFs, eEFs and eRFs play major roles in protein synthesis steps. Investigating the details of the eIF2 complex reveals that different eIF2 subunits play important roles in IBD. PRK plays an important part in inflammatorome activation and interacts with a variety of inflammatorome components, including NLRP3. The PERK-eIF2α-ATF4 signaling pathway is a potential therapeutic target for IBD. eIF2a was found overexpressed in gastrointestinal cancer. On the other hand, ATF4 plays a vital role in maintaining intestinal homeostasis, and the loss of eIF5B leads to increased translation of ATF4 transcripts through some mechanisms. There may be an association between eIF5B and IBD. eIFs may represent a new set of players associated with IBD and CRC, opening the door to an new area of GI tract research.

Changes in the expression of eIFs, eEFs and eRFs had been reported in a wide range of tumors, which played different roles in cell proliferation and tumorigenesis. Some of them may act as tumor suppressors, while others may contribute to the occurrence and progression of tumors. The effects of eIFs, eEFs and eRFs on CRC were shown in **Table 1**. These studies suggested that eIFs, eEFs and eRFs play a key role in CRC development and may be potential targets for CRC therapy.

**Table 1:** The eIFs, eEFs and eRFs play a role in CRC.

Factor	Function in CRC
eIF1	Overexpressed in CRC, oncogenic, linked to poor survival (14).
eIF2a	Overexpressed in CRC, important role in chemical-induced apoptosis and contributes to G2/M arrest (33).
eIF3a	Overexpressed in CRC, up-regulates normally translationally repressed proteins (41).
eIF3b	Overexpressed in CC, acts as oncogene in CC (44).
eIF3d	Play an oncogenic role in CC (49).
eIF3e	Overexpressed in CRC, interacts with the interferon-induced protein p56, plays potential integrated role in cell growth, development, and tumorigenesis (50).
eIF3g	Overexpressed in CRC, acts as oncogene, promotes metastasis and chemoresistance (54).
eIF3h	rs16892766 was identified as a CRC susceptibility SNP (55).
eIF3i	Proto-oncogene. The synthesis of COX-2 protein was up-regulated and the catenin/TCF4 signaling pathway was activated (56).
eIF3m	Overexpressed in CC, acts as oncogene, is linked with poor prognosis (57, 58).
eIF4A	Targeting MUC1-C with GO-203 inhibits the AKT-S6K-elF4A pathway by blocking cell proliferation and survival (67).
eIF4e	Over expressed in CRC, acts as oncogene, is linked with poor prognosis (68).
eIF5A	Overexpressed in CRC, acts as oncogene, is linked with poor prognosis (73).
eIF6	Overexpressed in CRC. Activates the multiple AKT-related cancer signaling pathways in CRC cells, thereby regulating cell proliferation, invasion, cell cycle and apoptosis (87).
eEF1	Overexpressed in CRC, acts as oncogene, promotes tumor growth and metastasis (99, 101).
eEF2	Overexpressed in CRC, acts as oncogene, Promotes G2/M progression and cell growth (104).
eEF-2K	Downregulated in CRC, acts as tumor suppressor, low expression is associated with short overall survival (108).

**Figure 2:**
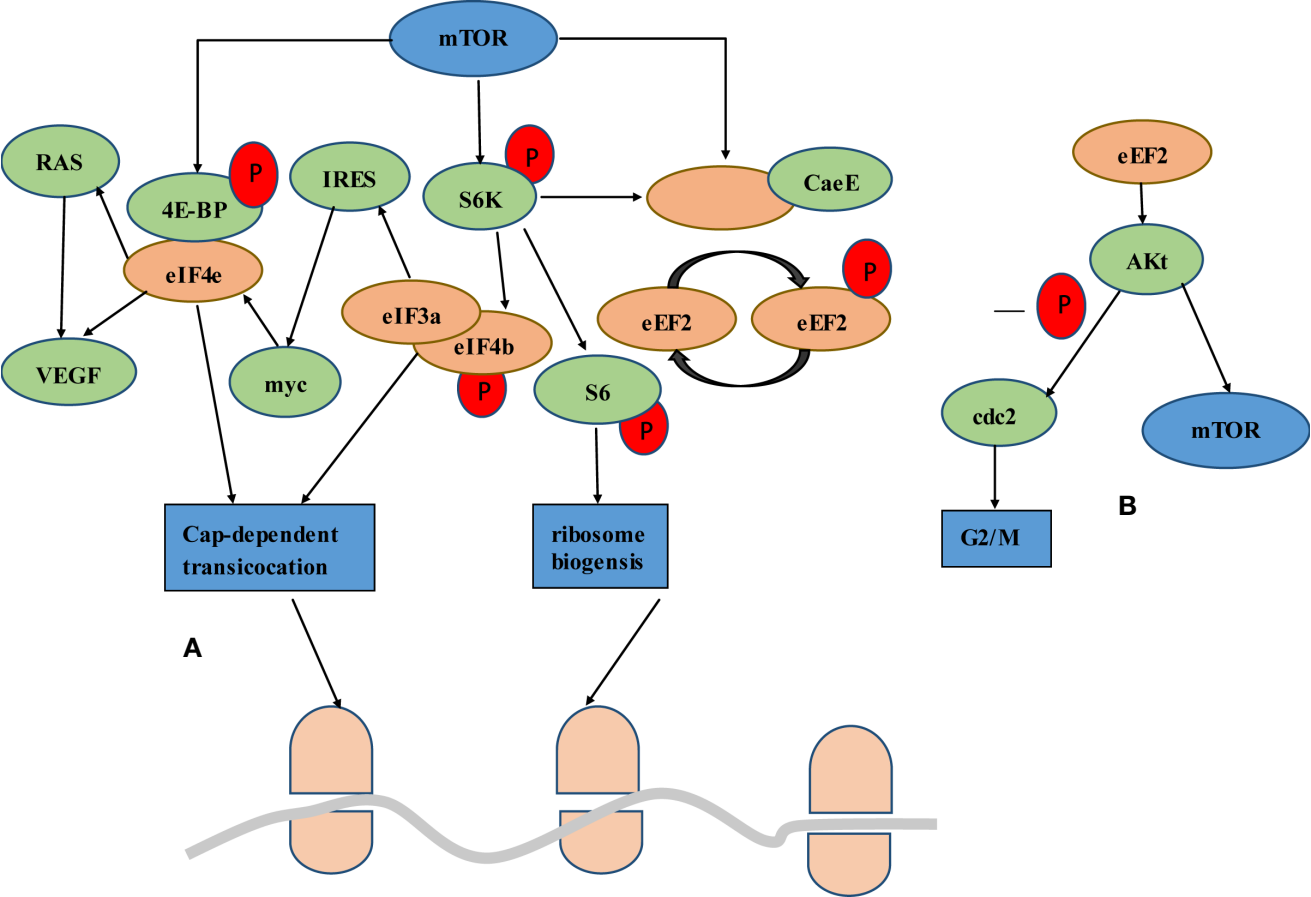
Interplay of mTOR signaling through protein translation. **(A)** The mTOR signal either goes through the 4eBP bound to eIF4e or through the S6K. mTOR phosphorylation of 4E-BP triggers the release of eIF4e and activates translation initiation. Upregulation of eIF4e triggers direct or Ras-induced VEGF translation. Activation of S6K leads to phosphorylation of eIF4b, which increases the binding affinity to eIF3a and thus promotes the translation of IRES dependent proteins such as myc. Myc in turn promotes the expression of eIF4e at the transcriptional level. S6K can affect the change of elongation. eEF2K is a negative regulator of eEF2, giving mTORC1 the ability to promote translation elongation through S6K. **(B)** Akt is a downstream molecule of eEF2, which regulates its activity. On the one hand, Akt activates the mTOR signaling pathway by inactivating the upstream regulator of mTOR, TSC2. On the other hand, Akt mediated eEF2 promotes G2/M progression through CDC2 activation.

mTOR, RAS signaling pathways and cell cycle regulation are critical for CRC development. RAS/MAPK and PI3K/AKT/mTOR pathways play key roles in promoting cell proliferation from membrane receptors to the nucleus. 4E-BP1 is one of the downstream molecules that receive signals from several intracellular pathways, including PI3K/AKT/mTOR and RAS/MAPK. 4E-BP1 is phosphorylated by AKT and MAPK. Phosphorylation induced by mTOR at 4E-BP triggers the release of eIF4e, enabling it to cooperate with the eIF4F complex and activate translation initiation. Vascular endothelial growth factor (VEGF) is a translation target for the upregulation of eIF4e, which is targeted directly or *via* Ras activation. VEGF is highly correlated with the neovascularization often observed in malignant tumors. Phosphorylation of eIF4b increases translation efficiency and binding affinity with eIF3a, which in turn promotes IRES dependent translation of proteins such as *myc*. Myc activates the transcription of eIF4e through a feedback mechanism, thereby increasing myc expression. The *APC* gene mutations had been observed in up to 80% of sporadic CRCs, and it plays a role in transcription regulation. Due to APC absent, c-myc expression was upregulated. In addition, mTORC1 had been shown to be an important downstream effector of Wnt signaling in the intestinal tract, while intestinal cells proliferation associated with Wnt signaling requires the mTORC1-S6K-eEF2K-eEF axis (**Figure 2**).

Recent research had found that dysregulation of protein translation may be one of the causes of cancer. Dysregulation translation results in abnormal gene expression, which was also found involved in cell proliferation or apoptosis, leading to abnormal cell growth and malignant transformation. Some abnormalities of the mRNA and protein levels of eIFs, eEFs and eRFs in colorectal diseases had been published. For example, eIF2a is overexpressed in IBD, most isoforms of eIFs and eEFs are overexpressed in CRC. In contrast, eEF-2K downregulation in CRC was associated with reduced overall survival.

Recent findings of protein translation based drugs, such as thymoquinone rapamycin, rapalogs and imatinib, may be available in the treatment of cancer. To sum up, these studies suggested that protein translation plays an important part in IBD and CRC development, and may be a potential therapeutic target for them.

## Author contributions

HCG made major contributions to the data analysis and manuscript writing. ZQ and ZXQ collected the data and participated in the manuscript revision. HR and DY collected the data. JH and YZH had the main primary idea and participated in the manuscript writing and revision. All authors contributed to the article and approved the submitted version.

## Funding

This work was financially supported by the project of department of science and Technology, Sichuan province (No.22ZDYF3780).

## Conflict of interest

The authors declare that the research was conducted in the absence of any commercial or financial relationships that could be construed as a potential conflict of interest.

## Publisher’s note

All claims expressed in this article are solely those of the authors and do not necessarily represent those of their affiliated organizations, or those of the publisher, the editors and the reviewers. Any product that may be evaluated in this article, or claim that may be made by its manufacturer, is not guaranteed or endorsed by the publisher.
